# Viral Factors in Modulation of Host Immune Response: A Route to Novel Antiviral Agents and New Therapeutic Approaches

**DOI:** 10.3390/ijms25179408

**Published:** 2024-08-29

**Authors:** Olga Tarasova, Anthi Petrou, Sergey M. Ivanov, Athina Geronikaki, Vladimir Poroikov

**Affiliations:** 1 Institute of Biomedical Chemistry, Moscow 119121, Russia; smivanov7@gmail.com (S.M.I.); vladimir.poroikov@ibmc.msk.ru (V.P.); 2 School of Pharmacy, Aristotle University of Thessaloniki, 541 24 Thessaloniki, Greece; anthi.petrou.thessaloniki1@gmail.com (A.P.); geronikaki@gmail.com (A.G.)

**Keywords:** viral infections, virus–host interactions, herpesviruses, hepatitis B, SARS-CoV-2, HIV, antiviral agents

## Abstract

Viruses utilize host cells at all stages of their life cycle, from the transcription of genes and translation of viral proteins to the release of viral copies. The human immune system counteracts viruses through a variety of complex mechanisms, including both innate and adaptive components. Viruses have an ability to evade different components of the immune system and affect them, leading to disruption. This review covers contemporary knowledge about the virus-induced complex interplay of molecular interactions, including regulation of transcription and translation in host cells resulting in the modulation of immune system functions. Thorough investigation of molecular mechanisms and signaling pathways that are involved in modulating of host immune response to viral infections can help to develop novel approaches for antiviral therapy. In this review, we consider new therapeutic approaches for antiviral treatment. Modern therapeutic strategies for the treatment and cure of human immunodeficiency virus (HIV) are considered in detail because HIV is a unique example of a virus that leads to host T lymphocyte deregulation and significant modulation of the host immune response. Furthermore, peculiarities of some promising novel agents for the treatment of various viral infections are described.

## 1. Introduction

Understanding of the molecular mechanisms of host response to infection allows researchers to develop new therapeutic strategies, including pathogenesis-directed therapy and the usage of drugs that are able to reduce severe complications. Viruses are capable of evading immune response through various mechanisms that include direct interaction with human immune cells or dysregulation of various intracellular signaling pathways. Evasion usually means that a virus is able to avoid elimination by the host’s immune system [1]. Most viruses are known to evade immune response for successful implementation of various life cycle stages including entry, replication, translation, and release of viral copies. Along with mechanisms that involve viral envelope and structural proteins, important mechanisms of immune response regulation also include the influence of viral proteases on the host pathways controlling immune processes. For instance, papain-like proteases of both severe acute respiratory syndrome coronaviruses 1 and 2 (SARS-CoV and SARS-CoV-2) suppress interferon type 1 production and NF-kappa B signaling pathways and antagonize innate immunity by the cleavage of ubiquitin and downregulation of interferon-stimulating genes, respectively [2,3,4,5]. Most viruses, including herpes viruses, hepatitis B, and human immunodeficiency virus (HIV) are able to suppress components of both innate and adaptive immune response. Various viruses use different molecular mechanisms to evade innate immune response. Most of these mechanisms are associated with the inhibition of signaling pathways involved in viral replication in host cells and with the influence on some other cascades that control interferon production [2,3,4,5]. The mechanisms that viruses utilize to evade adaptive immune response are closely associated with two common tactics and include the development of latency and processes leading to exhaustion of T cells. In this review, we consider the ways used by various viruses for evading both innate and adaptive immune responses, and the promising approaches for designing new drugs and therapeutic strategies for treatment of viral infections. We highlight the studies relevant to the analysis of viral impact on host immune systems with the recent experimental information obtained in this field published in the last decade.

## 2. Molecular Mechanisms of Immune Response to Viral Infection

A virus enters the cell through direct interactions with the host cell membrane. Proteins and other host cell molecules (the so-called host-dependency factors) are required for viral replication [6,7,8]. The virus hijacks host cell signaling and metabolic pathways to produce viral copies. Viruses can use the host cell for replication through interactions between viral and host proteins and RNA in the cell. The cell has the so-called host restriction factors that interfere with viral replication. The innate immune response is then activated, and cytokines are secreted in response to the viral infection. After the innate immune response, the adaptive component is activated. At this stage, viral proteins and microRNAs can act on various signaling pathways to evade host defense mechanisms. Chronic infection leads to a prolonged and less effective immune response, which is associated with immune exhaustion.

**Figure 1:**
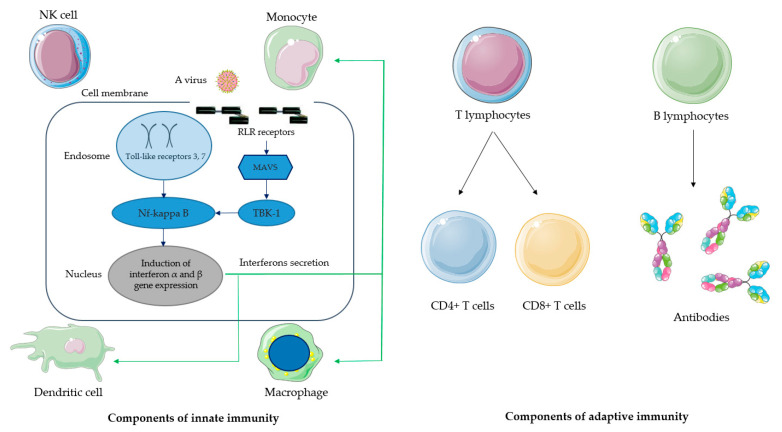
The main components of innate and adaptive immune responses.

Viral proteins can interfere with both innate and adaptive components of the immune response (Figure 1, Table 1). Since there is an interplay between the innate and adaptive immunity, it can be expected that systemic effects will be mediated by both these components [9]. Despite the fact that a much data has been collected to date, most of specific mechanisms of immune evasion and corresponding key proteins still require clarification [1].

Innate immune response (Figure 1) includes responses controlled by Toll-like receptors (TLRs), RIG-1 receptors (RLRs), signaling pathways, and the TANK-binding kinase 1 pathway. TLRs are expressed in dendritic cells and macrophages (they can also be found in fibroblasts and epithelial cells) and are involved in the activation of NK cells. The TLR pathway is of high significance for innate immunity because it controls production of cytokines (tumor necrosis factor (TNF), interleukin 1, interleukin 6) and interferon type 1 (α and β). The recognition of pathogen-associated molecular patterns (PAMPs) can be processed by TLRs either on the cellular membrane or in endosomes. RLRs belong to a family of DExD/H box RNA helicases that recognize PAMPs of viral RNA [10]. RLRs control downstream regulation of interferon type 1 (IFN1) production. TANK-binding kinase 1 (TBK1) is an essential component of antiviral immune response. It controls the expression of IFN1 (α and β). The modulation of TBK1 is considered a potential option for therapy of viral infections [11].

The effects of some viruses (e.g., hepatitis C, SARS-CoV-2, adenovirus) on the host immune response include modulation of the innate immune response, which in turn is closely associated to their influence on the adaptive response, mainly through activation of cytokines, e.g., interleukins and tumor necrosis factor alpha [12,13,14,15,16,17] and the importance of the innate immune response for antibody production [18]. At the same time, molecular mechanisms associated with the influence of viruses on innate immunity that are important for their effects on the adaptive immune system are under investigation [18].

Adaptive components of the immune response include mainly T and B lymphocytes, which are capable of initiating a specific immune response against a particular viral antigen, followed by elimination of the infected cells. B lymphocytes are a source of antibodies. T lymphocytes are divided into CD4+ T lymphocytes, which have the helper function, including activation and suppression of immune system cells [19]. CD8+ T lymphocytes have an effector function; they are killers of infected cells [20].

**Table 1:** Common and unique molecular mechanisms that some viruses utilize to evade host immune response.

Virus	Key Viral Enzyme	Molecular Mechanism	Systemic Effect	Reference
SARS-CoV-2	Papain-like protease (PLpro)	PLpro cleaves ubiquitin-like interferon-stimulated gene 15 protein (ISG15)	Deregulation of host interferon response	[1,21]
	NSP1, NSP6	Inhibition of STAT1 phosphorylation antagonizes the IFN signaling	Inhibition of interferon response	[22]
	NSP8	Interaction with MDA5 leads to the inhibition of IFN3 phosphorylation	Inhibition of interferon response	[22]
Hepatitis B	HBc, HBs, HBe antigens	Exhaustion of HBV specific T and B cells. Hepatotropism	Immune tolerance occurring due to HBc, HBs and HBe antigens	[1]
	IP0, ICP4, US3	ICP0-mediated translocation of USP7 (Ubiquitin-specific-processing protease7) from the nucleus to cytoplasm	Inhibition of TLR-mediated immune response	[23,24]
Herpes simplex virus type 1	US11	US11 interacts with RIG-1 and MDA5 and leads to a blockade of signal transduction	Negative downstream regulation of interferon type 1 transcription	[24]
	RNA intermediates, host derived RNAs	RNA intermediates interact with RLRs, leading to inhibition of signal transduction	Negative downstream regulation of interferon type 1 transcription	[24]
	ICP0	ICP0 mediates modulation of interferon type 3 response	Inhibition of interferon response	[24]
	HSV gE and gC	HSV gE and gC inhibits specific antibody response	Inhibition of antibody response	[25,26]
Kaposi’s Sarcoma-Associated Herpes virus	ORF64	ORF64 interferes with RIG-I ubiquitination	Inhibition of interferon response	[27]
Epstein–Barr virus	BPLF1	BPLF1 interferes with RIG-I ubiquitination and downregulates interferon response; BPLF1 interacts with and deubiquitinates TRAF6	Inhibition of interferon response	[28,29,30]
Influenza A virus	NS1, NS2	NS1 inhibits RIG-1 activation; NS2 interacts with IRF7	Inhibition of interferon production	[31]
	PB1	Interaction with MAVS	Inhibition of interferon production	[31]
	PB2, HA	Inhibition of JAK1/STAT pathway	Inhibition of interferon signaling	[31]
Human immuno-deficiency virus	Nef	HIV Nef protein interacts with naïve B cells and macrophages and inhibits interferon response	Inhibition of interferon response	[32]
	Vif	HIV Vif inhibits ubiquitination of STING influencing on STING-TBK1-IRF3 pathway	Inhibition of the production of type I interferon	[33]

The novel coronavirus, SARS-CoV-2, has multiple mechanisms that provide immune evasion through the proteases [34]. One such mechanism is based on the interaction of papain-like protease (PLPro) with ubiquitin-like interferon-stimulated gene 15 protein (ISG15) and its cleavage, leading to a deubiquitylating activity. ISG15 is an activator of interferon (IFN) type 1 and 2 release [35,36] and it affects the maturation of dendritic cells, NK cells, and T lymphocytes. ISG15 transcription is activated in viral infections [23]. The effect of ISG15 on release of IFN1 and IFN2 is mediated by its binding with the LFA1 receptor on the surface of NK cells and T lymphocytes.

Along with viral proteases, some important mechanisms of immune system modulation by SARS-CoV-2 include inhibition of STAT1 phosphorylation by NSP1 and NSP6 proteins that antagonize the interferon signaling [22] leading to suppression of the IFN-β and IFN3 production [22]. The detailed description of SARS-CoV2-mediated modulation of immune response is provided in the reviews by Farooq Rashid et al. [22], Teresa I Ng et al. [37], and Evgenii Gusev et al. [38].

Herpes viruses are able to disrupt innate immune response through interfering with TLR and RLR pathways [29,30]. The effect of herpes viruses on the adaptive immune system is under active investigation [39,40]. Mechanisms that herpes viruses use to evade the adaptive immune system include latency and some specific ways to depress T cell receptor signal transduction and T cell cytolytic activity [39,40].

It should be noted that some mechanisms of effects on adaptive immune response are mediated by direct interactions of a virus with the host cell membrane, in most cases, leading to systemic effects. For instance, the HBe antigen of the hepatitis B virus is responsible for the HBV-specific immune tolerance of T cells produced in the thymus (the so-called central tolerance) [41]. The immune tolerance allows viruses to escape from the human immune system due to high structural similarity between viral antigens and host cell proteins. The mechanisms of immune tolerance of hepatitis B virus have a great importance due to its hepatotropism and hepatotoxicity, leading to chronic inflammation, cirrhosis, fibrosis and carcinoma [1], but the mechanisms of innate immune response evasion are not studied in detail nowadays and there is still a room for improvement [1].

The human immunodeficiency virus has several mechanisms that allows for escaping the host’s immune response and affecting CD4+ and CD8+ T cells. The usage of immunodominant variable loops, incomplete binding surfaces, oligomeric occlusion, glycosylation, conformation masking, and multivalent interactions is described as a common strategy of HIV escape from neutralization by anti-HIV broadly neutralizing antibodies [42,43,44]. HIV typically leads to abnormalities of both T lymphocyte and B-lymphocyte function that result in the dysfunction of innate and adaptive immune responses. Such effects can be reached through both direct interaction of HIV virions with the host cell membrane and systemic effects caused by HIV infection.

One of the mechanisms that is currently being studied is the interaction of HIV viral infectivity factor (Vif) with the host stimulator of interferon genes (STING), resulting in the inhibition of type I interferon production [33,45].

Direct interaction of bound virions with FCR+ and CD21+ B cells, α4β7+ B cells and follicular dendritic cells is associated with inhibition of B cell responses, while HIV Nef protein interferes with the function of naïve B cells and macrophages [32]. HIV infection also causes systemic effects that may include an increase in immune activation that is associated with activation of plasmablasts, activated memory and tissue-like memory cells [32]. This process is closely connected with elevated levels of inflammatory cytokines and type 1 interferons, and is associated with increased activation of both T and B cells and immune cell exhaustion [32,46,47]. These processes lead to lymphoid tissue hyperplasia, which, in turn, corresponds to early loss of morphologic integrity and disrupts antibody response [32,46,47]. The altered homeostasis and regulation of B cells compartments results in the decreased pool of naïve B cells and increased apoptosis. Mechanisms of system-level effects of HIV-infection on immune system deregulation are being studied actively nowadays.

The latency is another common mechanism that allows viruses to evade the host’s immune response [39,40,48,49,50,51]. The latency is associated with the persistence of a virus with restricted expression of viral genes [48]. The thorough investigation of viral latency, as well as other mechanisms of immune evasion by viruses, is helpful for development of novel potential therapeutic strategies for antiviral treatments and cures.

Deep understanding of the molecular mechanisms of immune evasion contributes to the development of antiviral agents with activity against multiple viral infections (so-called “pan-antivirals”), because several viruses use similar mechanisms that lead to the suppression of the interferon response. Such a strategy may be beneficial in cases of resistance to direct antivirals. As viral evasion can suppress host immunity, this mechanism may lead to the development of co-infections including cancer [52] and neurological complications [53,54,55]. Inhibition of viral evasion will therefore be useful in co-infection prevention, in particular, bacterial infections, which can develop within several days after viral invasion. In chronic viral infections, overcoming immune evasion may be important for cancer prevention. The study of immune evasion mechanisms can also be helpful in predicting potential side effects of chemical compounds that may interact with specific proteins involved in the adaptive and innate immune pathways as off-targets.

## 3. Development of Therapeutic Strategies Based on the Current Knowledge on Virus–Host Interactions and Modulation of Immune Response to Viral Infections

Along with the small-molecule inhibitors that exhibit direct antiviral effects [56,57,58,59,60,61,62,63,64,65,66], some novel therapeutic strategies engaged in the specific components of immune response are continuously being investigated for various viruses. Such strategies can be beneficial for the development of agents active against a variety of viruses [65] or specific antiviral agents against a particular virus to prevent complications [67,68]. We summarize a few recently developed strategies of viral infection treatment affecting host immune response in Table 2.

In the study by J. Chen et al. [69], the authors found a key kinase of the antiviral pathway, TBK1, that promotes METTL3 activation and m^6^A (N^6^-methyladenosine) mRNA modification to stabilize interferon 3 (IRF3) mRNA that influences interferon production. The role of N^6^-methyladenosine in the virus–host interaction and viral infection progression as shown earlier [70] allows for suggesting that METTL3-mediated activation of M^6^A may be considered a promising target for antiviral therapy. Further, the inhibitors of methyltransferase-like 3 (METTL3), which is responsible for catalyzing N6-methyladenosine (m^6^A) modification on mRNA, were described as potential inhibitors of viral infections [71,72,73]. The study by R. Winkler and co-authors explains the inhibitory effect against METTL3 on the innate immune response, which is associated with possible induction of interferon-stimulated genes [74]. Therefore, the search for inhibitors of METTL3 or those that interfere with METTL3 activation represents one of the novel strategies to cure viral infections through activation of innate immunity.

The small-molecule inhibitors of METTL3 as agents with potential antiviral activity against SARS-CoV and SARS-CoV-2 were described in the study by H.M. Burgess et al. [72]. Recently, a series of new nucleoside analogues have been designed to act as inhibitors of human METTL3 [80]. The authors of the study showed that the series of novel chemical compounds developed on the basis of the previously developed METTL3 inhibitors methylthioadenosine (MTA) and S-tubercidinyl-l-homocysteine (TubHcy) exhibited activity against enterovirus 71 (EV-71) in cell-based assays. Interestingly, the authors used structure-based design and proposed hit-to-lead modifications, taking into account the SAM binding pocket of METTL3, followed by the docking procedure. Most of the designed chemical compounds were characterized by micromolar activities against EV-71 in cell-based assays.

In addition to stimulating the immune response by activating interferon production, strategies aimed at downregulating pro-inflammatory cytokines were evaluated for COVID-19 treatment as a potential method of stabilizing the anti-inflammatory response [75,76,77,78]. In particular, the antiviral agents with potential anti-inflammatory effect were developed as potential drug candidates for COVID-19 therapy, including nitazoxanide, sabizabulin, opaganib, selinexor, atazanavir, etc. [77] and evaluated in clinical trials. Interestingly, nitazoxanide has previously been shown to have antiviral activity against hepatitis B and C viruses and influenza, in addition to its anti-SARS-CoV-2 activity [77]. Nitazoxanide reached phase IV clinical trials and was considered a promising drug for the treatment of COVID-19 [78]. Given that nitazoxanide may have anti-inflammatory effect in addition to antiviral activity, it may be important for the treatment of viral infections characterized by chronic inflammation, such as hepatitis C virus infection [78]. Combination therapy involving the use of antiviral and anti-inflammatory drugs in COVID-19 therapy is also considered a promising strategy for COVID-19 treatment [76,78].

The recent study by M. Tsuji et al. [79] describes the action of the glycolipid 7DW8-5 on virus replication in host organisms. The control of viral infection is achieved by binding of 7DW8-5 to CD1d on antigen-presenting cells, leading to the release of cytokines and chemokines, which in turn results in an antiviral effect.

Hepatitis B antiviral therapy, which includes the administration of nucleoside analogues and pegylated interferon α, has some limitations due to the unlimited duration of treatment and side effects, respectively. These limitations have led to the development of new strategies that include the application of small interfering RNA for post-translational control of the hepatitis B virus [81,82]. Other strategies include targeting host factors involved in the development of hepatitis B infection, including agents that can bind host dependency factors responsible for viral entry. Examples of such host factors that are considered targets for novel antiviral agents are heparan sulfate molecules on the cell surface or sodium taurocholate transporting polypeptide (NTCP) [83]. Two recently developed strategies involving small molecules include application of Toll-like receptor agonists that lead to induction of type I interferon response [81] and immune checkpoint inhibitors [107], the molecules that inhibit the receptors overexpressed by T lymphocytes, including cytotoxic T lymphocyte-associated protein 4 (CTLA-4) and the programmed cell death 1 (PD-1) protein.

Some studies are focused on small-molecule inhibitors of the proteins that regulate the activity of both innate and adaptive components of immune response [65]. Small-molecule inhibitors of human ubiquitin-specific protease 7 (USP7), which downregulates the antiviral activity mediated by interferon type 1, are considered to be the agents that improve antiviral immune response and can be used as a boost therapy along with IFN1 [65]. Small-molecule inhibitors of the BPLF1 Epstein–Barr virus enzyme, including the top inhibitory compound, suramin, which may increase the RIG-I-mediated interferon response along with inhibition of NF-κB signaling, were described in the study by Sage L. Atkins et al. [28]. BPLF1 interacts with TRAF6, which, in turn, leads to the inhibition of NF-κB signaling during infection [28]. Therefore, despite the fact that inhibitors of BPLF1 may be considered to be rather direct-acting antivirals, they can have an effect on the host immune system as well [28]. In the study by Sage L. Atkins, it was shown that suramin also inhibits ORF64, a Kaposi Sarcoma herpes virus homolog of deubiquitinating enzyme BPLF1 [28].

In the study by Kelsey M. Haas et al. [68], the authors identified 332 protein–protein interactions between influenza A viruses and 13 host-modulated kinases, including MAP2K3, MAP2K6, CDK2, ILK, PRKDC, etc., using affinity purification-mass spectrometry (AP-MS). The authors mentioned that modulated proteins may be involved in RNA splicing and processing, cellular and nuclear membranes, regulation of gene silencing, and innate immune response. Experimental testing of the 37 host–protein targeting compounds provides the possibility of finding 16 compounds suppressing replication of influenza A virus (various strains), some of which had also activity against SARS-CoV-2. The most potent compounds included daunorubicin, verdinexor, selinexor, bafilomycin A1, alexidine, lestaurtinib, and MRT68921. Although a particular mechanism of viral replication through interaction of a chemical compound with host proteins may not be identified yet for all tested compounds, such results help to shed light on the possibility of using host targets modulators in antiviral therapy.

In general, an analysis of the therapeutic strategies that are based on the development of host-directed acting antivirals shows that antiviral drugs that may interact with host proteins can be classified into two sub-groups. The first group includes inhibitors or activators of the particular host proteins that may be involved in the viral entry or viral replication process, and the second group consists of the chemical compounds that may act on the components of innate or adaptive antiviral immunity [108].

The pharmacological agents that can modulate the host immune response to a wide range of viral infections have certain advantages and limitations. The most important advantage is their potential to suppress the production of several different viruses that have similar mechanisms of immune evasion, such as an effect on the particular components of the RLR pathway. It may also be useful in the case of de novo viral infections that are rapidly spreading in the human population, where the lack of information on their structure and function may require considerable time and effort to develop a compound with direct antiviral mechanisms of action. Significant experience with drug repurposing in the search for potential host-directed antivirals with anti-inflammatory activity was gained during the COVID-19 pandemic. The anti-inflammatory effect in combination with antiviral activity is another potential advantage of therapeutic strategies based on modulation of the host immune response.

At the same time, the indirect mechanism of antiviral action may be a reason for lower efficacy of drugs that may act on certain components of the immune response compared to direct-acting antivirals. Potential adverse effects and toxicities should be carefully evaluated when direct or indirect modulation of host proteins is utilized in the development of antiviral strategies.

The efficacy of these new strategies and an understanding of their possible limitations is the subject of thorough investigation.

## 4. New Approaches to HIV Treatment and Cure Based on Fighting Latency and Targeting CD4+ T Lymphocyte Proliferation

HIV is one of the key viruses to consider in the search for therapeutic approaches based on knowledge of viral factors that modulate the host immune response. Its importance in the identification of potential therapeutic strategies is related to its ability to disrupt both innate and adaptive components of the immune response. Novel strategies for curing HIV infection are of high significance due to the ability of the virus to influence the function of both CD4+ and CD8+ T lymphocytes, and its latency. Since latency is one of the important issues in treatment of viral infections, the efforts of researchers are focused on curing latent infections. There are several directions in the search for new approaches to HIV therapy (Table 2).

Most existing HIV drugs are inhibitors of three HIV enzymes, namely protease, reverse transcriptase, and integrase; however, researchers have also found the approaches for modulating the activity of molecules that mediate HIV entry into CD4-containing host cells, including chemokine C receptor type 5 (CCR5) and C-X-C chemokine receptor type 4 (CXCR4), as significant targets [87,88,89,90,91]. In most HIV-infected individuals, this receptor is CCR5 [90]. The approved drug maraviroc is an antagonist of CCR5, and ibalizumab is a monoclonal antibody to the epitope at the CD4 receptor. Maraviroc may be involved in the downregulation of HIV-associated inflammation [91,109] and immune activation [109]. Ibalizumab was shown to restore CD4+ T cell function [110]. Therefore, these drugs act as direct antivirals and may have an impact on the host immune system.

Several other strategies have been reported in the literature to develop active molecules that target factors mediating virus entry into the human cell [111].

One of the important strategies to inhibit HIV-1 replication is the inhibition of interaction between HIV-1 viral infectivity factor (Vif) and Apolipoprotein B mRNA-editing catalytic polypeptide-like 3 family members (APOBEC3), which is the host restriction factor inhibiting HIV replication. HIV-1 Vif has an impact of the degradation of APOBEC3 though the activity of core-binding factor β (CBFβ) of the E3 ubiquitin ligase complex. Therefore, prevention of Vif interaction with CBFβ will lead to the release of APOBEC3 and will result in inhibition of HIV-1 replication [93]. The chemical compound CV-3 with the mentioned mechanism of action and activity on the HIV replication in micromolar concentration is described in the study conducted by S. Duan et al. [93].

Another direction in the development of new therapeutic strategies for the treatment of HIV infection is the search for molecules that potentially regulate cellular processes resulting from the entry of HIV into the cell and its integration into the human genome in order to affect these molecules. In particular, such molecules include master regulators, proteins, and genes at the top of the regulatory protein interaction networks that can affect the expression of a number of genes. The search for such molecules in relation to HIV infection has been developing over the past few years. For example, the study by M. Haneklaus et al., 2013 [92] examines the role of miR-223 small interfering RNA (miRNA) in inflammation, viral infections, and cancer. In particular, since miR-223 expression has been shown to be reduced in influenza virus-induced infection, hepatitis B, and type 2 diabetes, leukemia, and lymphoma, this may contribute to a reduction in oncogenic transformation of bone marrow cells. Target proteins of miR-223 that may exhibit anti-inflammatory effects in viral infections include granzyme B, IKKα, Roquin, and STAT3. In CD4+ T cells where HIV replication does not occur, miR-223 expression is elevated compared to levels in CD4+ T lymphocytes where HIV replication is activated. The physiological significance of this observation is currently under investigation. Another study demonstrated that in macrophages, expression of miR-223 and other cellular factors that inhibit HIV infection could be induced by type I interferon, which might explain its antiviral activity [112].

The signaling pathway controlled by mTORC1 as a master regulator (mammalian target of rapamycin complex 1) is considered one of the mechanisms regulating intracellular signaling in HIV infection. Specifically, CD4+ T cells, which are the primary target of HIV-1, are cells that can differentiate into various effector lines (Th1, Th2, Th17) in response to various inflammatory cytokines. The signaling pathway controlled by mTORC1 aids this differentiation process by promoting glycolysis and lipid biosynthesis [113,114,115]. It has been shown that mTORC1-deficient T cells have a limited ability to differentiate [113]. T cells in the absence of the mTORC1 activator RHEB also fail to differentiate into Th1 and Th17 [113]. In addition, deletion of the mTORC1 inhibitor, TSC1, results in increased mTORC1 activity, leading to increased Th1 and Th17 differentiation and inflammation in mice [116,117].

CD8+ T cells are capable of destroying cells with markers of tumor transformation or cells infected with viruses (effector cytotoxic T cells) and maintaining long-term memory (memory T cells). Generation of CD8+ effector T cells is thought to be regulated by mTORC1 activity [118,119,120]. The role of mTORC1 in the regulation of HIV latency has also been shown. Thus, the strategy of modulation of mTORC1-controlled pathway components is being investigated as one of the possible therapies for HIV infection.

The principle of the “shock and kill” approach is to activate lymphocytes infected with HIV in the latent stage and apply antiretroviral therapy to destroy the latent HIV reservoirs [121]. Usage of various inhibitors affecting intracellular pathways, including histone deacetylase, histone methyltransferase, DNA methyltransferase, bromodomain inhibitors, and protein kinase C (PKC) agonists, was investigated [94,95,96,97,98,99,100,101,121,122]. The clinical trials demonstrated that use of histone deacetylase inhibitors (vorinostat, panobinostat, romidepsin, and disulfiram) increased production of cell-associated HIV RNA and/or viremia in plasma after administration [94,95,96,97,98,99,100,101,121,122]. However, the application of the aforementioned drugs did not result in a significant reduction in the volume of the latent HIV reservoir [101]. The “shock and kill” approach includes attempts to use the immune checkpoint inhibitors, leading to the reduced effector function of CD8+ T lymphocytes. In particular, ipilimumab (CTLA-4 inhibitor) and nivolumab (PD-1 inhibitor) are used to upregulate CD8+ T cell function to combat HIV latency by restoring its replication and T cell exhaustion [95,96]. Immune checkpoint inhibitors are applied for HIV-infected people with cancer; however, their usage in anti-HIV treatment is still under discussion [97]. It should be noted that the “shock and kill” approach has several major limitations, including insufficient host immune response against cells in which the viral genome is expressed, the presence of mutations in the virus in the latency state, or insufficient scale of latency reversal [122]. However, the main limitation, as noted by T.A. Rasmussen and co-authors, is the inability to accurately estimate the amount of latent HIV reservoir to be reversed [122]. This seems to be related to the fact that the “shock and kill” strategy is not widespread in clinical practice.

The “block and lock” approach is based on the use of latency-promoting agents that help “lock” the virus promoter and “block” the transcription of the virus [102,103]. This strategy can be advantageous for a number of reasons, including the possible duration of the effect [102] and, according to some authors, the lack of need for highly active antiretroviral therapy, HAART [102,103]. A limitation of this approach is that the use of “block and lock” does not lead to complete elimination of the virus; however, on the other hand, this is also a limitation of HAART.

The search for drugs aimed at regulating the expression of individual receptors that can lead to decrease in mitochondrial depletion in CD4+ T lymphocytes is considered an additional therapeutic strategy in the treatment of HIV infection. In this case, the drugs target the activation of CD4+ T lymphocyte proliferation. In particular, bezafibrate induces expression of PPARG coactivator 1 alpha (PGC1α) through the PPAR-responsive element in its promoter region [104,105,106]. GW7647 and pioglitazone are PPARα and PPARγ agonists, respectively [104]. Bezafibrate, GW7647, and pioglitazone were tested on CD4+ T cells [104]. As a result, their ability to induce the foxp3 gene in regulatory CD4+ T cells and a role in mitochondrial biogenesis were demonstrated [104]. Bezafibrate, GW7647, and pioglitazone failed to induce either PGC1α expression or cell proliferation in CD4+ T cell samples from patients who had no long-term increase in CD4+ T cell levels. However, exposure to interleukin-15 (IL-15) induced PGC1α expression through activation of the mTOR pathway in some of the patients taking antiretroviral therapy with long-term low CD4+ T lymphocyte levels. Indeed, IL-15 has been shown to induce mTOR activation [106], which promotes PGC1α expression via the transcription factor Yin Yang 1 (YY1). The findings suggest that IL-15 induced PGC1α and promoted restoration of CD4+ T cell proliferation.

More recently, lenacapavir (Sunlenca^®^) has been developed, a novel, first-in-class, picomolar, long-acting HIV-1 capsid inhibitor [123,124,125,126,127,128,129,130]. Its efficacy in the treatment of multidrug-resistant HIV-1 infection has been demonstrated in clinical trials [127,128,129]. Although lenacapavir has a favorable metabolic and safety profile [130], its widespread clinical use may help to identify a whole range of opportunities and limitations in HIV treatment, including its use in combination with other antiretroviral drugs and those used to treat co-morbidities.

In addition to creating new HIV enzyme inhibitors and molecules that affect the regulation of HIV interaction with the human body, several other approaches to treat HIV infection are currently under development, namely anti-HIV vaccines [131] and immunotherapy using neutralizing antibodies [132]. The difficulty in creating an anti-HIV vaccine is associated with the fact that people do not develop protective immunity to HIV infection, whereas almost all successful vaccines have been created for diseases for which immunity can be developed after exposure to a live pathogen [133]. Nevertheless, the development of effective anti-HIV vaccines is under active investigation, and novel approaches continue to develop [134,135].

## 5. Recent Efforts in the Development of Monoclonal Antibodies in Treatment of Viral Infections

The approaches for the development of antibodies against viral infections have been recently overviewed by Giuseppe Pantaleo and co-authors [136]. Antibodies are macromolecules that contain two antigen-binding domain fragments (Fabs) that bind to and neutralize pathogens. They are connected to the crystallizable fragment (Fc) domain by a hinge region, providing certain conformational flexibility. Nowadays, the main efforts in antibody development are associated with the usage of hybridoma technology [137] and B-cells from convalescent patients infected with SARS-CoV-2 [138,139,140,141,142,143,144].

The development of highly potent monoclonal antibodies is part of a general trend, along with the generation of polyclonal antibodies or cocktails of monoclonal and bispecific antibodies [145]. Generation of monoclonal antibodies was of particular importance during the coronavirus (COVID-19) pandemic due to its emergence and the lack of potent chemical compounds that can be used as a basis for the development of novel antiviral agents. At the same time, recently, production of monoclonal antibodies inhibiting neuraminidase enzyme activity was reported in the study by Lena Hansen and co-authors [146]. The authors describe two monoclonal antibodies obtained from a patient reconvalescent from H1N1 virus infection. Antibodies exhibited inhibitory activity of H1N1 and swine N1 viruses.

Recent efforts in the development of monoclonal antibodies for fighting with viral infections are presented in Table 3.

Palivizumab represents an example of a monoclonal antibody approved and applied in clinical practice against the respiratory syncytial virus (RSV) [147]. Pediatric infection caused by RSV is a major cause of complicated respiratory illnesses in infants, especially neonates and premature newborns. Two antibodies, nirsevimab and MK-1654, are also used for immunization of children against infection caused by RSV [147,148].

The development of novel antibodies against SARS-CoV-2 [139,140,141,142,143,144] and the Ebola virus [149] have been recently presented, providing possibilities of use this knowledge for future development of monoclonal antibodies. At the same time, application of antibody cocktails for protection against multiple viral infections is one of several novel recently developed strategies for fighting co-infections [149]. An example of such a strategy is reported in the study conducted by Guodong Liu and co-authors [149], where two monoclonal antibodies, 4F9 and 6H8, were generated using hybridoma technology, tested in cell-free bioassays, and shown to be effective against the Ebola and Sudan viruses in animal models.

Another novel strategy is the development of cross-protective antibodies. An example of such an approach is provided in the study by Madelyn Caban et al. [150], where the authors focused on the discovery and characterization of two cross-neutralizing monoclonal antibodies. One of the reported antibodies, 3 × 1, was effective against both human parainfluenza virus types one and three viruses, and another one, MxR, targeted both RSV and human metapneumovirus correspondingly. One may assume that the approaches aimed at targeting a pair of several viruses with high homology in proteins and infecting predominantly the same organ or system of the human body will be one of the most promising and developing approaches in the future, as it may help to develop antiviral therapies against newly emerging viruses relatively quickly.

Nivolumab (PD-1 inhibitor) is used as a checkpoint inhibitor in the treatment of HIV infection [95,96] and hepatitis B [107], and ipilimumab (CTLA-4 inhibitor) is used in therapy of HIV infection [95,96].

One of the main advantages of monoclonal antibodies in the treatment of viral infections is their efficacy and the possibility of prolonged effect observed as a result of their use. A possible limitation of monoclonal antibodies is viral evasion of antibody-induced humoral immunity due to specific mutations in viral proteins; such mutations have been described for the SARS-CoV-2 receptor-binding domain of the spike protein [151]. In this context, the investigation of viral escaping of host adaptive immunity in patients after passive immunization with monoclonal antibodies or after vaccine therapy may be relevant for future studies.

## 6. Conclusions

The development and severity of viral infections are associated with the host immune response to viral infections. Therefore, the thorough investigation of the particular molecular mechanisms that use viruses to evade the immune response can be useful in the search for new therapeutic agents, including those that can express non-specific antiviral activity. Such novel therapeutic strategies are associated with modulation of the innate immunity pathways, including modulation of the RIG-1 receptor family, inhibition of ubiquitin-specific protease 7, and the search for METTL3 inhibitors that downregulate M^6^A mRNA modification, which, in turn, induce interferon response.

Some specific therapeutic approaches that can be used to treat particular viral infections are under active development. The interest in activating the antiviral immune response in the development of appropriate strategies and novel potential antivirals is linked to the possibility of identifying compounds that are active against multiple viruses, including resistant variants, due to modulation of the host rather than the viral targets. At the same time, such antivirals should have low adverse and toxic effects, which would require additional preclinical studies and multiple stages of clinical trials, potentially limiting the development of antivirals that may interact with host proteins.

Small-molecule inhibitors of BPLF1, an Epstein–Barr virus enzyme, and its Kaposi sarcoma herpes virus homolog, ORF64, may have an effect on the RLR pathway, followed by induction of the interferon I response. Such compounds, although acting on viral proteins, may therefore have an indirect mechanism of action to enhance the antiviral immune response. Such therapeutic strategies may therefore be of interest for future research and development.

For human immunodeficiency virus, along with the “gold standard” highly active antiretroviral therapy, researchers have developed some approaches aimed at the prevention of the virus binding with co-receptors on the CD4+ T cells, overcoming the latency, including “block and shock” and “shock and kill” approaches. Despite the fact that these new approaches have demonstrated efficacy, they are not used in wide clinical practice yet. Due to unique HIV properties that are associated with the ability to cause pathological activation of T cells, leading to their apoptosis, the development of new approaches directed at the reactivation of CD4+ T lymphocytes are of interest for the scientific community. Lenacapavir, a picomolar inhibitor of the HIV-1 capsid, is one of the most promising agents for the treatment and cure of HIV, and its use in widespread clinical practice is expected to provide new insights into possible therapeutic approaches for patients with HIV infection and its co-morbidities. Chemical agents capable of inhibiting the interactions of HIV-1 Vif with APOBEC3 protein core binding factor β are another promising strategy for the development of potential antiviral agents with an indirect mechanism of action and an effect on the host immune system. In general, the development of pharmacological agents that prevent viral evasion of the host immune system and have an anti-inflammatory effect may be beneficial for therapeutic purposes.

SARS-CoV-2 uses papain-like protease for downregulation of host interferon response; therefore, some inhibitors of this immune-related mechanism can help interfere with both key components of viral life cycle and immune deregulation caused by SARS-CoV-2. The search for potential antivirals that can both interfere with viral targets and modulate an immune response may be one of the most promising strategies for developing new drugs that are effective against viral infections.

At the same time, because the immune system involves multiple signaling cascades and its modulation by pharmacological agents can have a range of complex consequences, all novel agents should be carefully analyzed and their potential side effects and toxicities determined before use in clinical practice. In general, deeper understanding of immune-related mechanisms of host response to viral infections can be rather helpful for the development of novel therapeutic approaches for viral infections and for finding potential associations between viral infections and other diseases.

## Figures and Tables

**Table 2:** Therapeutic strategies based on the current knowledge on virus–host interactions.

Virus	Key Host Target	Drug	Effect	Reference
SARS-CoV-2	TBK1, METTL3	quercetin, nucleoside analogues	Stabilization of interferon 3 (IRF3) mRNA that influence interferon production	[1,69,70,71,72,73,74]
	Cytokines: TNF-α, IL-3, IL-6.	sabizabulin, opaganib, selinexor, atazanavir, nitazoxanide	Stabilizing of inflammatory response/antiviral effect	[22,75,76,77,78]
	CD1d	7DW8-5	Stimulation of NKT cells to release the cascade of cytokines and chemokines/inhibition of virus replication	[79]
Enterovirus-71	METTL3	methylthioadenosine & S-tubercidinyl-l-homocysteine analogs	Inhibition of METTL3	[80]
Hepatitis B	TLRs 3,7,8,9	GS-9620	Induction of peripheral IFN-stimulated gene 15 (ISG15) expression	[81,82]
	NTCP	myrcludex	binding to NTCP and blockade of de novo HBV infection	[83]
Herpes simplex virus type 1	Wnt/β-catenin	iCRT14 KYA1797K	Inhibition of β-catenin-dependent transcription/enhancement of the formation of the β-catenin destruction complex. Inhibition of virus penetration and virus replication	[84,85,86]
Kaposi’s Sarcoma-Associated Herpes virus	NF-κB signaling	suramin	Inhibitors of ORF64 prevent inhibition of TLR-mediated activation of NF-κB signaling, preventing inhibition of interferon response	[28]
Epstein–Barr virus	USP7/RIG family, NF-κB signaling, TRAF6	suramin	Inhibitors of BPLF1 prevent inhibition of TLR-mediated activation of NF-κB signaling, preventing inhibition of interferon response	[28]
Influenza A	MAP2K3, MAP2K6, CDK2, ILK, PRKDC	daunorubicin, verdinexor, selinexor, bafilomycin A1, alexidine, lestaurtinib, MRT68921	RNA splicing and processing, cellular and nuclear membranes, regulation of gene silencing, and innate immune response	[68]
Human immuno-deficiency virus	CCR5	maraviroc	Antagonists of CCR5 prevent entry of HIV into the host cell	[87,88,89,90,91]
	Granzyme B, IKKα, Roquin and STAT3	miR-223	Prevention of chronic inflammation	[92]
	CBFβ/Vif-3 complex	CV-3	inhibition of HIV-1 replication	[93]
Human immunodeficiency virus	Histone deacetylase/Histone methyltransferase, DNA methyltransferase, bromodomain inhibitors; protein kinase C (PKC) agonists	vorinostat, panobinostat, romidepsin, disulfiram,	“shock and kill” Activation of lymphocytes infected with HIV in the latent stage for further application of antiretroviral therapy	[94,95,96,97,98,99,100,101]
	LEDGF/p75 FACT HSP90 Jak-STAT BRD4 mTOR	didehydro-cortistatin A (dCA), LEDGINs curaxin CBL0100, AUY922, 17-AAG ruxolitinib, tofacitinib, ZL0580 PF-3758309, danusertib, AZ628, P276-00	“block and lock”, prevention of HIV transcription and reactivation in latently infected cells	[102,103]
	PPARα and PPARγ agonists	bezafibrate, GW7647, pioglitazone	Activation of CD4+ T lymphocytes proliferation	[104,105,106]

**Table 3:** Monoclonal antibodies in the therapy of viral infections.

Virus	Drug	Molecular Target	Reference
SARS-CoV-2	tixagevimab, cilgavimab	SARS-CoV-2 spike protein	[138,139,140,141,142,143,144]
Respiratory syncytial virus (RSV)	palivisumab, nirsevimab, MK-1654	An epitope of the F protein of RSV	[147,148]
Ebola virus, Sudan virus	4F9, 6H8	Ebola virus glycoprotein Sudan virus glycoprotein	[149]
Human parainfluenza virus	3 × 1	F protein of HPIV3	[150]
Respiratory syncytial virus Human pneumovirus	M×R	F protein of RSV	[150]
Hepatitis B infection (Immune checkpoint inhibitor)	nivolumab	Programmed cell death (PD-1) inhibitor	[107]
Human Immunodeficiency virus 1 (Immune checkpoint inhibitor)	nivolumab, ipilimumab	Cytotoxic T lymphocyte associated protein 4 (CTLA-4)	[95,96,97]

## References

[1] Kuipery A., Gehring A.J., Isogawa M. (2020). Mechanisms of HBV Immune Evasion. Antivir. Res..

[2] Devaraj S.G., Wang N., Chen Z., Chen Z., Tseng M., Barretto N., Lin R., Peters C.J., Tseng C.-T.K., Baker S.C. (2007). Regulation of IRF-3-Dependent Innate Immunity by the Papain-like Protease Domain of the Severe Acute Respiratory Syndrome Coronavirus. J. Biol. Chem..

[3] Frieman M., Ratia K., Johnston R.E., Mesecar A.D., Baric R.S. (2009). Severe Acute Respiratory Syndrome Coronavirus Papain-like Protease Ubiquitin-like Domain and Catalytic Domain Regulate Antagonism of IRF3 and NF-KappaB Signaling. J. Virol..

[4] Shin D., Mukherjee R., Grewe D., Bojkova D., Baek K., Bhattacharya A., Schulz L., Widera M., Mehdipour A.R., Tascher G. (2020). Papain-like Protease Regulates SARS-CoV-2 Viral Spread and Innate Immunity. Nature.

[5] Locci M., Havenar-Daughton C., Landais E., Wu J., Kroenke M.A., Arlehamn C.L., Su L.F., Cubas R., Davis M.M., Sette A. (2013). Human Circulating PD-1+CXCR3-CXCR5+ Memory Tfh Cells Are Highly Functional and Correlate with Broadly Neutralizing HIV Antibody Responses. Immunity.

[6] Ohlson M.B., Eitson J.L., Wells A.I., Kumar A., Jang S., Ni C., Xing C., Buszczak M., Schoggins J.W. (2023). Genome-Scale CRISPR Screening Reveals Host Factors Required for Ribosome Formation and Viral Replication. mBio.

[7] Fu C., Yang S., Yang X., Lian X., Huang Y., Dong X., Zhang Z. (2020). Human Gene Functional Network-Informed Prediction of HIV-1 Host Dependency Factors. mSystems.

[8] Ivanov S., Lagunin A., Filimonov D., Tarasova O. (2020). Network-Based Analysis of OMICs Data to Understand the HIV-Host Interaction. Front. Microbiol..

[9] Sun L., Wang X., Saredy J., Yuan Z., Yang X., Wang H. (2020). Innate-Adaptive Immunity Interplay and Redox Regulation in Immune Response. Redox Biol..

[10] Loo Y.-M., Gale M. (2011). Immune Signaling by RIG-I-like Receptors. Immunity.

[11] Zhao C., Zhao W. (2019). TANK-Binding Kinase 1 as a Novel Therapeutic Target for Viral Diseases. Expert. Opin. Ther. Targets.

[12] Soto J.A., Gálvez N.M.S., Andrade C.A., Pacheco G.A., Bohmwald K., Berrios R.V., Bueno S.M., Kalergis A.M. (2020). The Role of Dendritic Cells During Infections Caused by Highly Prevalent Viruses. Front. Immunol..

[13] Gelmez M.Y., Oktelik F.B., Tahrali I., Yilmaz V., Kucuksezer U.C., Akdeniz N., Cetin E.A., Kose M., Cinar C., Oguz F.S. (2022). Immune Modulation as a Consequence of SARS-CoV-2 Infection. Front. Immunol..

[14] Gadotti A.C., de Castro Deus M., Telles J.P., Wind R., Goes M., Garcia Charello Ossoski R., de Padua A.M., de Noronha L., Moreno-Amaral A., Baena C.P. (2020). IFN-γ Is an Independent Risk Factor Associated with Mortality in Patients with Moderate and Severe COVID-19 Infection. Virus Res..

[15] Trevejo J.M., Marino M.W., Philpott N., Josien R., Richards E.C., Elkon K.B., Falck-Pedersen E. (2001). TNF-α-Dependent Maturation of Local Dendritic Cells Is Critical for Activating the Adaptive Immune Response to Virus Infection. Proc. Natl. Acad. Sci. USA.

[16] Kumar A., Coquard L., Herbein G. (2016). Targeting TNF-Alpha in HIV-1 Infection. Curr. Drug Targets.

[17] Stuart J.D., Salinas E., Grakoui A. (2021). Immune System Control of Hepatitis C Virus Infection. Curr. Opin. Virol..

[18] Iwasaki A., Medzhitov R. (2015). Control of Adaptive Immunity by the Innate Immune System. Nat. Immunol..

[19] Luckheeram R.V., Zhou R., Verma A.D., Xia B. (2012). CD4^+^T Cells: Differentiation and Functions. Clin. Dev. Immunol..

[20] Koh C.-H., Lee S., Kwak M., Kim B.-S., Chung Y. (2023). CD8 T-Cell Subsets: Heterogeneity, Functions, and Therapeutic Potential. Exp. Mol. Med..

[21] Zhang M., Li J., Yan H., Huang J., Wang F., Liu T., Zeng L., Zhou F. (2021). ISGylation in Innate Antiviral Immunity and Pathogen Defense Responses: A Review. Front. Cell Dev. Biol..

[22] Rashid F., Xie Z., Suleman M., Shah A., Khan S., Luo S. (2022). Roles and Functions of SARS-CoV-2 Proteins in Host Immune Evasion. Front. Immunol..

[23] Daubeuf S., Singh D., Tan Y., Liu H., Federoff H.J., Bowers W.J., Tolba K. (2009). HSV ICP0 Recruits USP7 to Modulate TLR-Mediated Innate Response. Blood.

[24] Zhu H., Zheng C. (2020). The Race between Host Antiviral Innate Immunity and the Immune Evasion Strategies of Herpes Simplex Virus 1. Microbiol. Mol. Biol. Rev..

[25] Lubinski J.M., Jiang M., Hook L., Chang Y., Sarver C., Mastellos D., Lambris J.D., Cohen G.H., Eisenberg R.J., Friedman H.M. (2002). Herpes Simplex Virus Type 1 Evades the Effects of Antibody and Complement in Vivo. J. Virol..

[26] Collins W.J., Johnson D.C. (2003). Herpes Simplex Virus GE/GI Expressed in Epithelial Cells Interferes with Cell-to-Cell Spread. J. Virol..

[27] Inn K.-S., Lee S.-H., Rathbun J.Y., Wong L.-Y., Toth Z., Machida K., Ou J.-H.J., Jung J.U. (2011). Inhibition of RIG-I-Mediated Signaling by Kaposi’s Sarcoma-Associated Herpesvirus-Encoded Deubiquitinase ORF64. J. Virol..

[28] Atkins S.L., Motaib S., Wiser L.C., Hopcraft S.E., Hardy P.B., Shackelford J., Foote P., Wade A.H., Damania B., Pagano J.S. (2020). Small Molecule Screening Identifies Inhibitors of the Epstein-Barr Virus Deubiquitinating Enzyme, BPLF1. Antivir. Res..

[29] Gupta S., Ylä-Anttila P., Callegari S., Tsai M.-H., Delecluse H.-J., Masucci M.G. (2018). Herpesvirus Deconjugases Inhibit the IFN Response by Promoting TRIM25 Autoubiquitination and Functional Inactivation of the RIG-I Signalosome. PLoS Pathog..

[30] Lui W.-Y., Bharti A., Wong N.-H.M., Jangra S., Botelho M.G., Yuen K.-S., Jin D.-Y. (2023). Suppression of cGAS- and RIG-I-Mediated Innate Immune Signaling by Epstein-Barr Virus Deubiquitinase BPLF1. PLoS Pathog..

[31] Rashid F., Xie Z., Li M., Xie Z., Luo S., Xie L. (2023). Roles and Functions of IAV Proteins in Host Immune Evasion. Front. Immunol..

[32] Moir S., Fauci A.S. (2017). B-Cell Responses to HIV Infection. Immunol. Rev..

[33] Wang Y., Qian G., Zhu L., Zhao Z., Liu Y., Han W., Zhang X., Zhang Y., Xiong T., Zeng H. (2022). HIV-1 Vif Suppresses Antiviral Immunity by Targeting STING. Cell Mol. Immunol..

[34] Majerová T., Konvalinka J. (2022). Viral Proteases as Therapeutic Targets. Mol. Aspects Med..

[35] Chu H., Chan J.F.-W., Wang Y., Yuen T.T.-T., Chai Y., Hou Y., Shuai H., Yang D., Hu B., Huang X. (2020). Comparative Replication and Immune Activation Profiles of SARS-CoV-2 and SARS-CoV in Human Lungs: An Ex Vivo Study With Implications for the Pathogenesis of COVID-19. Clin. Infect. Dis..

[36] Hadjadj J., Yatim N., Barnabei L., Corneau A., Boussier J., Smith N., Péré H., Charbit B., Bondet V., Chenevier-Gobeaux C. (2020). Impaired Type I Interferon Activity and Inflammatory Responses in Severe COVID-19 Patients. Science.

[37] Ng T.I., Correia I., Seagal J., DeGoey D.A., Schrimpf M.R., Hardee D.J., Noey E.L., Kati W.M. (2022). Antiviral Drug Discovery for the Treatment of COVID-19 Infections. Viruses.

[38] Gusev E., Sarapultsev A., Solomatina L., Chereshnev V. (2022). SARS-CoV-2-Specific Immune Response and the Pathogenesis of COVID-19. Int. J. Mol. Sci..

[39] Zhu S., Viejo-Borbolla A. (2021). Pathogenesis and Virulence of Herpes Simplex Virus. Virulence.

[40] Sloan D.D., Han J.-Y., Sandifer T.K., Stewart M., Hinz A.J., Yoon M., Johnson D.C., Spear P.G., Jerome K.R. (2006). Inhibition of TCR Signaling by Herpes Simplex Virus. J. Immunol..

[41] Tian Y., Kuo C., Akbari O., Ou J.J. (2016). Maternal-Derived Hepatitis B Virus e Antigen Alters Macrophage Function in Offspring to Drive Viral Persistence after Vertical Transmission. Immunity.

[42] Dimitrov D.S. (2004). Virus Entry: Molecular Mechanisms and Biomedical Applications. Nat. Rev. Microbiol..

[43] Wei X., Decker J.M., Wang S., Hui H., Kappes J.C., Wu X., Salazar-Gonzalez J.F., Salazar M.G., Kilby J.M., Saag M.S. (2003). Antibody Neutralization and Escape by HIV-1. Nature.

[44] Kwong P.D., Doyle M.L., Casper D.J., Cicala C., Leavitt S.A., Majeed S., Steenbeke T.D., Venturi M., Chaiken I., Fung M. (2002). HIV-1 Evades Antibody-Mediated Neutralization through Conformational Masking of Receptor-Binding Sites. Nature.

[45] Zhang L., Li S., Xu X., Ma C., Zhang P., Ji W., Liu X. (2024). HIV-1 P17 Matrix Protein Enhances Type I Interferon Responses through the P17-OLA1-STING Axis. J. Cell Sci..

[46] Moir S., Ho J., Malaspina A., Wang W., DiPoto A.C., O’Shea M.A., Roby G., Kottilil S., Arthos J., Proschan M.A. (2008). Evidence for HIV-Associated B Cell Exhaustion in a Dysfunctional Memory B Cell Compartment in HIV-Infected Viremic Individuals. J. Exp. Med..

[47] Levesque M.C., Moody M.A., Hwang K.-K., Marshall D.J., Whitesides J.F., Amos J.D., Gurley T.C., Allgood S., Haynes B.B., Vandergrift N.A. (2009). Polyclonal B Cell Differentiation and Loss of Gastrointestinal Tract Germinal Centers in the Earliest Stages of HIV-1 Infection. PLoS Med..

[48] Speck S.H., Ganem D. (2010). Viral Latency and Its Regulation: Lessons from the Gamma-Herpesviruses. Cell Host Microbe.

[49] Murata T., Sugimoto A., Inagaki T., Yanagi Y., Watanabe T., Sato Y., Kimura H. (2021). Molecular Basis of Epstein-Barr Virus Latency Establishment and Lytic Reactivation. Viruses.

[50] Kennedy P.G.E., Mogensen T.H., Cohrs R.J. (2021). Recent Issues in Varicella-Zoster Virus Latency. Viruses.

[51] Dahabieh M.S., Battivelli E., Verdin E. (2015). Understanding HIV Latency: The Road to an HIV Cure. Annu. Rev. Med..

[52] Ameya G., Birri D.J. (2023). The Molecular Mechanisms of Virus-Induced Human Cancers. Microb. Pathog..

[53] Clé M., Eldin P., Briant L., Lannuzel A., Simonin Y., Van de Perre P., Cabié A., Salinas S. (2020). Neurocognitive Impacts of Arbovirus Infections. J. Neuroinflamm..

[54] Reiss A.B., Greene C., Dayaramani C., Rauchman S.H., Stecker M.M., De Leon J., Pinkhasov A. (2023). Long COVID, the Brain, Nerves, and Cognitive Function. Neurol. Int..

[55] Elendu C., Aguocha C.M., Okeke C.V., Okoro C.B., Peterson J.C. (2023). HIV-Related Neurocognitive Disorders: Diagnosis, Treatment, and Mental Health Implications: A Review. Medicine.

[56] Wang X., Zou P., Wu F., Lu L., Jiang S. (2017). Development of Small-Molecule Viral Inhibitors Targeting Various Stages of the Life Cycle of Emerging and Re-Emerging Viruses. Front. Med..

[57] Liang R., Wang L., Zhang N., Deng X., Su M., Su Y., Hu L., He C., Ying T., Jiang S. (2018). Development of Small-Molecule MERS-CoV Inhibitors. Viruses.

[58] Picazo E., Giordanetto F. (2015). Small Molecule Inhibitors of Ebola Virus Infection. Drug Discov. Today.

[59] Haese N., Powers J., Streblow D.N. (2020). Small Molecule Inhibitors Targeting Chikungunya Virus. Current Topics in Microbiology and Immunology.

[60] Ivanenkov Y.A., Aladinskiy V.A., Bushkov N.A., Ayginin A.A., Majouga A.G., Ivachtchenko A.V. (2017). Small-Molecule Inhibitors of Hepatitis C Virus (HCV) Non-Structural Protein 5A (NS5A): A Patent Review (2010–2015). Expert. Opin. Ther. Pat..

[61] Lu L., Yu F., Cai L., Debnath A.K., Jiang S. (2016). Development of Small-Molecule HIV Entry Inhibitors Specifically Targeting Gp120 or Gp41. Curr. Top. Med. Chem..

[62] Belda O., Targett-Adams P. (2012). Small Molecule Inhibitors of the Hepatitis C Virus-Encoded NS5A Protein. Virus Res..

[63] Elseginy S.A., Massarotti A., Nawwar G.A., Amin K.M., Brancale A. (2014). Small Molecule Inhibitors of West Nile Virus. Antivir. Chem. Chemother..

[64] Pitts J., Hsia C.-Y., Lian W., Wang J., Pfeil M.-P., Kwiatkowski N., Li Z., Jang J., Gray N.S., Yang P.L. (2019). Identification of Small Molecule Inhibitors Targeting the Zika Virus Envelope Protein. Antivir. Res..

[65] Yuan Y., Miao Y., Zeng C., Liu J., Chen X., Qian L., Wang X., Qian F., Yu Z., Wang J. (2020). Small-Molecule Inhibitors of Ubiquitin-Specific Protease 7 Enhance Type-I Interferon Antiviral Efficacy by Destabilizing SOCS1. Immunology.

[66] Ibba R., Corona P., Nonne F., Caria P., Serreli G., Palmas V., Riu F., Sestito S., Nieddu M., Loddo R. (2023). Design, Synthesis, and Antiviral Activities of New Benzotriazole-Based Derivatives. Pharmaceuticals.

[67] Bugatti K., Sartori A., Battistini L., Coppa C., Vanhulle E., Noppen S., Provinciael B., Naesens L., Stevaert A., Contini A. (2023). Novel Polymyxin-Inspired Peptidomimetics Targeting the SARS-CoV-2 Spike:hACE2 Interface. Int. J. Mol. Sci..

[68] Haas K.M., McGregor M.J., Bouhaddou M., Polacco B.J., Kim E.-Y., Nguyen T.T., Newton B.W., Urbanowski M., Kim H., Williams M.A.P. (2023). Proteomic and Genetic Analyses of Influenza A Viruses Identify Pan-Viral Host Targets. Nat. Commun..

[69] Chen J., Wei X., Wang X., Liu T., Zhao Y., Chen L., Luo Y., Du H., Li Y., Liu T. (2022). TBK1-METTL3 Axis Facilitates Antiviral Immunity. Cell Rep..

[70] Dang W., Xie Y., Cao P., Xin S., Wang J., Li S., Li Y., Lu J. (2019). N6-Methyladenosine and Viral Infection. Front. Microbiol..

[71] Du Y., Yuan Y., Xu L., Zhao F., Wang W., Xu Y., Tian X. (2022). Discovery of METTL3 Small Molecule Inhibitors by Virtual Screening of Natural Products. Front. Pharmacol..

[72] Burgess H.M., Depledge D.P., Thompson L., Srinivas K.P., Grande R.C., Vink E.I., Abebe J.S., Blackaby W.P., Hendrick A., Albertella M.R. (2021). Targeting the m6A RNA Modification Pathway Blocks SARS-CoV-2 and HCoV-OC43 Replication. Genes Dev..

[73] Kostyusheva A., Brezgin S., Glebe D., Kostyushev D., Chulanov V. (2021). Host-Cell Interactions in HBV Infection and Pathogenesis: The Emerging Role of m6A Modification. Emerg. Microbes Infect..

[74] Winkler R., Gillis E., Lasman L., Safra M., Geula S., Soyris C., Nachshon A., Tai-Schmiedel J., Friedman N., Le-Trilling V.T.K. (2019). m6A Modification Controls the Innate Immune Response to Infection by Targeting Type I Interferons. Nat. Immunol..

[75] Zagaliotis P., Petrou A., Mystridis G., Geronikaki A., Vizirianakis I., Walsh T. (2022). Developing New Treatments for COVID-19 through Dual-Action Antiviral/Anti-Inflammatory Small Molecules and Physiologically Based Pharmacokinetic Modeling. Int. J. Mol. Sci..

[76] Sasaki M., Sugi T., Iida S., Hirata Y., Kusakabe S., Konishi K., Itakura Y., Tabata K., Kishimoto M., Kobayashi H. (2024). Combination Therapy with Oral Antiviral and Anti-Inflammatory Drugs Improves the Efficacy of Delayed Treatment in a COVID-19 Hamster Model. eBioMedicine.

[77] Mahmoud D.B., Shitu Z., Mostafa A. (2020). Drug Repurposing of Nitazoxanide: Can It Be an Effective Therapy for COVID-19?. J. Genet. Eng. Biotechnol..

[78] Bharti C., Sharma S., Goswami N., Sharma H., Rabbani S.A., Kumar S. (2021). Role of Nitazoxanide as a Repurposed Drug in the Treatment and Management of Various Diseases. Drugs Today.

[79] Tsuji M., Nair M.S., Masuda K., Castagna C., Chong Z., Darling T.L., Seehra K., Hwang Y., Ribeiro Á.L., Ferreira G.M. (2023). An Immunostimulatory Glycolipid That Blocks SARS-CoV-2, RSV, and Influenza Infections in Vivo. Nat. Commun..

[80] Salerno M., Varricchio C., Bevilacqua F., Jochmans D., Neyts J., Brancale A., Ferla S., Bassetto M. (2023). Rational Design of Novel Nucleoside Analogues Reveals Potent Antiviral Agents for EV71. Eur. J. Med. Chem..

[81] Phillips S., Jagatia R., Chokshi S. (2022). Novel Therapeutic Strategies for Chronic Hepatitis B. Virulence.

[82] Dawood A., Abdul Basit S., Jayaraj M., Gish R.G. (2017). Drugs in Development for Hepatitis B. Drugs.

[83] Mitra B., Thapa R.J., Guo H., Block T.M. (2018). Host Functions Used by Hepatitis B Virus to Complete Its Life Cycle: Implications for Developing Host-Targeting Agents to Treat Chronic Hepatitis B. Antivir. Res..

[84] Harrison K.S., Jones C. (2021). Wnt Antagonists Suppress Herpes Simplex Virus Type 1 Productive Infection. Antivir. Res..

[85] Koujah L., Madavaraju K., Agelidis A.M., Patil C.D., Shukla D. (2021). Heparanase-Induced Activation of AKT Stabilizes β-Catenin and Modulates Wnt/β-Catenin Signaling during Herpes Simplex Virus 1 Infection. mBio.

[86] Zhu L., Jones C. (2018). The Canonical Wnt/β-Catenin Signaling Pathway Stimulates Herpes Simplex Virus 1 Productive Infection. Virus Res..

[87] Zhang C., Zhu R., Cao Q., Yang X., Huang Z., An J. (2020). Discoveries and Developments of CXCR4-Targeted HIV-1 Entry Inhibitors. Exp. Biol. Med..

[88] Armani-Tourret M., Zhou Z., Gasser R., Staropoli I., Cantaloube-Ferrieu V., Benureau Y., Garcia-Perez J., Pérez-Olmeda M., Lorin V., Puissant-Lubrano B. (2021). Mechanisms of HIV-1 Evasion to the Antiviral Activity of Chemokine CXCL12 Indicate Potential Links with Pathogenesis. PLoS Pathog..

[89] Mohamed H., Gurrola T., Berman R., Collins M., Sariyer I.K., Nonnemacher M.R., Wigdahl B. (2021). Targeting CCR5 as a Component of an HIV-1 Therapeutic Strategy. Front. Immunol..

[90] Weichseldorfer M., Affram Y., Heredia A., Tagaya Y., Benedetti F., Zella D., Reitz M., Romerio F., Latinovic O.S. (2020). Anti-HIV Activity of Standard Combined Antiretroviral Therapy in Primary Cells Is Intensified by CCR5-Targeting Drugs. AIDS Res. Hum. Retroviruses.

[91] Rossi R., Lichtner M., De Rosa A., Sauzullo I., Mengoni F., Massetti A.P., Mastroianni C.M., Vullo V. (2011). In Vitro Effect of Anti-Human Immunodeficiency Virus CCR5 Antagonist Maraviroc on Chemotactic Activity of Monocytes, Macrophages and Dendritic Cells. Clin. Exp. Immunol..

[92] Haneklaus M., Gerlic M., O’Neill L.A.J., Masters S.L. (2013). MiR-223: Infection, Inflammation and Cancer. J. Intern. Med..

[93] Duan S., Wang S., Song Y., Gao N., Meng L., Gai Y., Zhang Y., Wang S., Wang C., Yu B. (2020). A Novel HIV-1 Inhibitor That Blocks Viral Replication and Rescues APOBEC3s by Interrupting Vif/CBFβ Interaction. J. Biol. Chem..

[94] Rasmussen T.A., Tolstrup M., Brinkmann C.R., Olesen R., Erikstrup C., Solomon A., Winckelmann A., Palmer S., Dinarello C., Buzon M. (2014). Panobinostat, a Histone Deacetylase Inhibitor, for Latent-Virus Reactivation in HIV-Infected Patients on Suppressive Antiretroviral Therapy: A Phase 1/2, Single Group, Clinical Trial. Lancet HIV.

[95] Castelli V., Lombardi A., Palomba E., Bozzi G., Ungaro R., Alagna L., Mangioni D., Muscatello A., Bandera A., Gori A. (2021). Immune Checkpoint Inhibitors in People Living with HIV/AIDS: Facts and Controversies. Cells.

[96] Trautmann L., Janbazian L., Chomont N., Said E.A., Gimmig S., Bessette B., Boulassel M.-R., Delwart E., Sepulveda H., Balderas R.S. (2006). Upregulation of PD-1 Expression on HIV-Specific CD8+ T Cells Leads to Reversible Immune Dysfunction. Nat. Med..

[97] Abbar B., Baron M., Katlama C., Marcelin A.-G., Veyri M., Autran B., Guihot A., Spano J.-P. (2020). Immune Checkpoint Inhibitors in People Living with HIV: What about Anti-HIV Effects?. Aids.

[98] Archin N.M., Liberty A.L., Kashuba A.D., Choudhary S.K., Kuruc J.D., Crooks A.M., Parker D.C., Anderson E.M., Kearney M.F., Strain M.C. (2012). Administration of Vorinostat Disrupts HIV-1 Latency in Patients on Antiretroviral Therapy. Nature.

[99] Elliott J.H., McMahon J.H., Chang C.C., Lee S.A., Hartogensis W., Bumpus N., Savic R., Roney J., Hoh R., Solomon A. (2015). Short-Term Administration of Disulfiram for Reversal of Latent HIV Infection: A Phase 2 Dose-Escalation Study. Lancet HIV.

[100] Elliott J.H., Wightman F., Solomon A., Ghneim K., Ahlers J., Cameron M.J., Smith M.Z., Spelman T., McMahon J., Velayudham P. (2014). Activation of HIV Transcription with Short-Course Vorinostat in HIV-Infected Patients on Suppressive Antiretroviral Therapy. PLoS Pathog..

[101] Rasmussen T.A., Tolstrup M., Søgaard O.S. (2016). Reversal of Latency as Part of a Cure for HIV-1. Trends Microbiol..

[102] Moranguinho I., Valente S.T. (2020). Block-And-Lock: New Horizons for a Cure for HIV-1. Viruses.

[103] Ahlenstiel C.L., Symonds G., Kent S.J., Kelleher A.D. (2020). Block and Lock HIV Cure Strategies to Control the Latent Reservoir. Front. Cell. Infect. Microbiol..

[104] Younes S.A. (2022). Mitochondrial Exhaustion of Memory CD4 T-Cells in Treated HIV-1 Infection. Immunometabolism.

[105] Morris S.R., Chen B., Mudd J.C., Panigrahi S., Shive C.L., Sieg S.F., Cameron C.M., Zidar D.A., Funderburg N.T., Younes S.-A. (2020). Inflammescent CX3CR1+CD57+CD8+ T Cells Are Generated and Expanded by IL-15. JCI Insight.

[106] Cunningham J.T., Rodgers J.T., Arlow D.H., Vazquez F., Mootha V.K., Puigserver P. (2007). MTOR Controls Mitochondrial Oxidative Function through a YY1-PGC-1alpha Transcriptional Complex. Nature.

[107] Gardiner D., Lalezari J., Lawitz E., DiMicco M., Ghalib R., Reddy K.R., Chang K.-M., Sulkowski M., Marro S.O., Anderson J. (2013). A Randomized, Double-Blind, Placebo-Controlled Assessment of BMS-936558, a Fully Human Monoclonal Antibody to Programmed Death-1 (PD-1), in Patients with Chronic Hepatitis C Virus Infection. PLoS ONE.

[108] Kumar N., Sharma S., Kumar R., Tripathi B.N., Barua S., Ly H., Rouse B.T. (2020). Host-Directed Antiviral Therapy. Clin. Microbiol. Rev..

[109] Funderburg N., Kalinowska M., Eason J., Goodrich J., Heera J., Mayer H., Rajicic N., Valdez H., Lederman M.M. (2010). Effects of Maraviroc and Efavirenz on Markers of Immune Activation and Inflammation and Associations with CD4+ Cell Rises in HIV-Infected Patients. PLoS ONE.

[110] Iacob S.A., Iacob D.G. (2017). Ibalizumab Targeting CD4 Receptors, An Emerging Molecule in HIV Therapy. Front. Microbiol..

[111] Zhang D., Li W., Jiang S. (2015). Peptide Fusion Inhibitors Targeting the HIV-1 Gp41: A Patent Review (2009–2014). Expert. Opin. Ther. Pat..

[112] Cobos Jiménez V., Booiman T., de Taeye S.W., van Dort K.A., Rits M.A.N., Hamann J., Kootstra N.A. (2012). Differential Expression of HIV-1 Interfering Factors in Monocyte-Derived Macrophages Stimulated with Polarizing Cytokines or Interferons. Sci. Rep..

[113] Delgoffe G.M., Kole T.P., Zheng Y., Zarek P.E., Matthews K.L., Xiao B., Worley P.F., Kozma S.C., Powell J.D. (2009). The MTOR Kinase Differentially Regulates Effector and Regulatory T Cell Lineage Commitment. Immunity.

[114] Kurebayashi Y., Nagai S., Ikejiri A., Ohtani M., Ichiyama K., Baba Y., Yamada T., Egami S., Hoshii T., Hirao A. (2012). PI3K-Akt-MTORC1-S6K1/2 Axis Controls Th17 Differentiation by Regulating Gfi1 Expression and Nuclear Translocation of RORγ. Cell Rep..

[115] Yang K., Shrestha S., Zeng H., Karmaus P.W.F., Neale G., Vogel P., Guertin D.A., Lamb R.F., Chi H. (2013). T Cell Exit from Quiescence and Differentiation into Th2 Cells Depend on Raptor-MTORC1-Mediated Metabolic Reprogramming. Immunity.

[116] Park Y., Jin H.-S., Lopez J., Elly C., Kim G., Murai M., Kronenberg M., Liu Y.-C. (2013). TSC1 Regulates the Balance between Effector and Regulatory T Cells. J. Clin. Investig..

[117] Nakaya M., Xiao Y., Zhou X., Chang J.-H., Chang M., Cheng X., Blonska M., Lin X., Sun S.-C. (2014). Inflammatory T Cell Responses Rely on Amino Acid Transporter ASCT2 Facilitation of Glutamine Uptake and MTORC1 Kinase Activation. Immunity.

[118] Araki K., Turner A.P., Shaffer V.O., Gangappa S., Keller S.A., Bachmann M.F., Larsen C.P., Ahmed R. (2009). MTOR Regulates Memory CD8 T-Cell Differentiation. Nature.

[119] Pearce E.L., Walsh M.C., Cejas P.J., Harms G.M., Shen H., Wang L.-S., Jones R.G., Choi Y. (2009). Enhancing CD8 T-Cell Memory by Modulating Fatty Acid Metabolism. Nature.

[120] Rao R.R., Li Q., Odunsi K., Shrikant P.A. (2010). The MTOR Kinase Determines Effector versus Memory CD8+ T Cell Fate by Regulating the Expression of Transcription Factors T-Bet and Eomesodermin. Immunity.

[121] Kim Y., Anderson J.L., Lewin S.R. (2018). Getting the “Kill” into “Shock and Kill”: Strategies to Eliminate Latent HIV. Cell Host Microbe.

[122] Rasmussen T.A., Lewin S.R. (2016). Shocking HIV out of Hiding: Where Are We with Clinical Trials of Latency Reversing Agents?. Curr. Opin. HIV AIDS.

[123] Paik J. (2022). Lenacapavir: First Approval. Drugs.

[124] Dvory-Sobol H., Shaik N., Callebaut C., Rhee M.S. (2022). Lenacapavir: A First-in-Class HIV-1 Capsid Inhibitor. Curr. Opin. HIV AIDS.

[125] Beninger P. (2024). Lenacapavir. Clin. Ther..

[126] Tuan J., Ogbuagu O. (2023). Lenacapavir: A Twice-Yearly Treatment for Adults with Multidrug-Resistant HIV Infection and Limited Treatment Options. Expert. Rev. Anti-Infect. Ther..

[127] Ogbuagu O., Segal-Maurer S., Ratanasuwan W., Avihingsanon A., Brinson C., Workowski K., Antinori A., Yazdanpanah Y., Trottier B., Wang H. (2023). Efficacy and Safety of the Novel Capsid Inhibitor Lenacapavir to Treat Multidrug-Resistant HIV: Week 52 Results of a Phase 2/3 Trial. Lancet HIV.

[128] Tailor M.W., Chahine E.B., Koren D., Sherman E.M. (2024). Lenacapavir: A Novel Long-Acting Capsid Inhibitor for HIV. Ann. Pharmacother..

[129] Segal-Maurer S., DeJesus E., Stellbrink H.-J., Castagna A., Richmond G.J., Sinclair G.I., Siripassorn K., Ruane P.J., Berhe M., Wang H. (2022). Capsid Inhibition with Lenacapavir in Multidrug-Resistant HIV-1 Infection. N. Engl. J. Med..

[130] Di Perri G. (2023). Pharmacological Outlook of Lenacapavir: A Novel First-in-Class Long-Acting HIV-1 Capsid Inhibitor. Infez. Med..

[131] Hsu D.C., O’Connell R.J. (2017). Progress in HIV Vaccine Development. Hum. Vaccin. Immunother..

[132] Sok D., Burton D.R. (2018). Recent Progress in Broadly Neutralizing Antibodies to HIV. Nat. Immunol..

[133] Brett-Major D.M., Crowell T.A., Michael N.L. (2017). Prospecting for an HIV Vaccine. Trop. Dis. Travel. Med. Vaccines.

[134] Burton D.R. (2019). Advancing an HIV Vaccine; Advancing Vaccinology. Nat. Rev. Immunol..

[135] Hokello J., Sharma A.L., Tyagi M. (2021). An Update on the HIV DNA Vaccine Strategy. Vaccines.

[136] Pantaleo G., Correia B., Fenwick C., Joo V.S., Perez L. (2022). Antibodies to Combat Viral Infections: Development Strategies and Progress. Nat. Rev. Drug Discov..

[137] Mitra S., Tomar P.C. (2021). Hybridoma Technology; Advancements, Clinical Significance, and Future Aspects. J. Genet. Eng. Biotechnol..

[138] Ryu D.-K., Song R., Kim M., Kim Y.-I., Kim C., Kim J.-I., Kwon K.-S., Tijsma A.S., Nuijten P.M., van Baalen C.A. (2021). Therapeutic Effect of CT-P59 against SARS-CoV-2 South African Variant. Biochem. Biophys. Res. Commun..

[139] Westendorf K., Žentelis S., Wang L., Foster D., Vaillancourt P., Wiggin M., Lovett E., van der Lee R., Hendle J., Pustilnik A. (2022). LY-CoV1404 (Bebtelovimab) Potently Neutralizes SARS-CoV-2 Variants. Cell Rep..

[140] Focosi D., McConnell S., Casadevall A., Cappello E., Valdiserra G., Tuccori M. (2022). Monoclonal Antibody Therapies against SARS-CoV-2. Lancet Infect. Dis..

[141] Ordaya E.E., Razonable R.R. (2024). Emerging Anti-Spike Monoclonal Antibodies against SARS-CoV-2. Expert. Opin. Biol. Ther..

[142] ACTIV-3/Therapeutics for Inpatients with COVID-19 (TICO) Study Group (2022). Efficacy and Safety of Two Neutralising Monoclonal Antibody Therapies, Sotrovimab and BRII-196 plus BRII-198, for Adults Hospitalised with COVID-19 (TICO): A Randomised Controlled Trial. Lancet Infect. Dis..

[143] Sakharkar M., Rappazzo C.G., Wieland-Alter W.F., Hsieh C.-L., Wrapp D., Esterman E.S., Kaku C.I., Wec A.Z., Geoghegan J.C., McLellan J.S. (2021). Prolonged Evolution of the Human B Cell Response to SARS-CoV-2 Infection. Sci. Immunol..

[144] Levin M.J., Ustianowski A., De Wit S., Launay O., Avila M., Templeton A., Yuan Y., Seegobin S., Ellery A., Levinson D.J. (2022). Intramuscular AZD7442 (Tixagevimab-Cilgavimab) for Prevention of COVID-19. N. Engl. J. Med..

[145] Both L., Banyard A.C., van Dolleweerd C., Wright E., Ma J.K.-C., Fooks A.R. (2013). Monoclonal Antibodies for Prophylactic and Therapeutic Use against Viral Infections. Vaccine.

[146] Hansen L., McMahon M., Turner H.L., Zhu X., Turner J.S., Ozorowski G., Stadlbauer D., Vahokoski J., Schmitz A.J., Rizk A.A. (2023). Human Anti-N1 Monoclonal Antibodies Elicited by Pandemic H1N1 Virus Infection Broadly Inhibit HxN1 Viruses in Vitro and in Vivo. Immunity.

[147] Esposito S., Abu Raya B., Baraldi E., Flanagan K., Martinon Torres F., Tsolia M., Zielen S. (2022). RSV Prevention in All Infants: Which Is the Most Preferable Strategy?. Front. Immunol..

[148] Langedijk A.C., Harding E.R., Konya B., Vrancken B., Lebbink R.J., Evers A., Willemsen J., Lemey P., Bont L.J. (2022). A Systematic Review on Global RSV Genetic Data: Identification of Knowledge Gaps. Rev. Med. Virol..

[149] Liu G., He S., Chan M., Zhang Z., Schulz H., Cao W., Rahim M.N., Audet J., Garnett L., Wec A. (2023). A Pan-Ebolavirus Monoclonal Antibody Cocktail Provides Protection Against Ebola and Sudan Viruses. J. Infect. Dis..

[150] Cabán M., Rodarte J.V., Bibby M., Gray M.D., Taylor J.J., Pancera M., Boonyaratanakornkit J. (2023). Cross-Protective Antibodies against Common Endemic Respiratory Viruses. Nat. Commun..

[151] Waldmann H. (2019). Human Monoclonal Antibodies: The Benefits of Humanization. Methods Mol. Biol..

